# Mechanical Behaviour of Geopolymer Concretes with Foamed Geopolymer and Lightweight Mineral Aggregates for Chimney Flue Elements

**DOI:** 10.3390/ma19091811

**Published:** 2026-04-29

**Authors:** Michał Łach, Agnieszka Przybek, Maria Hebdowska-Krupa, Wojciech Franus, Maciej Szeląg, Krzysztof Krajniak, Adam Masłoń

**Affiliations:** 1 Faculty of Material Engineering and Physics, Cracow University of Technology, Jana Pawła II 37, 31-864 Cracow, Poland; agnieszka.przybek@pk.edu.pl (A.P.); maria.hebdowska-krupa@pk.edu.pl (M.H.-K.); 2 Interdisciplinary Center for Circular Economy, Cracow University of Technology, Warszawska 24, 31-155 Cracow, Poland; 3 CUT Doctoral School, Cracow University of Technology, Warszawska 24, 31-155 Cracow, Poland; 4 Department of Construction Materials Engineering and Geoengineering, Lublin University of Technology, Nadbystrzycka 40, 20-618 Lublin, Poland; w.franus@pollub.pl (W.F.); maciej.szelag@pollub.pl (M.S.); 5 BRATA Limited Liability Company, Bałtycka 56, 76-211 Objazda, Poland; emag@brata.pl; 6 Department of Environmental Engineering and Chemistry, Rzeszow University of Technology, Powstańców Warszawy 12 Av., 35-029 Rzeszów, Poland

**Keywords:** geopolymer concretes, lightweight aggregates, mechanical strength, thermal insulation, chimney liners

## Abstract

Geopolymer concretes are increasingly regarded as advanced construction materials for applications requiring high thermal and chemical resistance. This article is a continuation of previously published research and focuses on the mechanical behaviour of geopolymer concretes containing aggregates made of foamed geopolymers and lightweight mineral aggregates, such as expanded clay and perlite, intended for use in chimney flue components. The aim of the study was to determine the influence of lightweight aggregates on the relationship between thermal insulation and the strength parameters of geopolymer concretes intended for use at elevated temperatures. Foamed geopolymer aggregates were produced by a controlled chemical foaming process, followed by grinding to specific grain sizes, yielding highly porous aggregates with low thermal conductivity, reaching approximately 0.075–0.099 W/(m·K). These aggregates were used as lightweight fillers in geopolymer concretes based on class F fly ash activated with alkaline solutions. The resulting composites were designed to combine low density and high thermal insulation with adequate mechanical strength. The mechanical properties of the developed concretes were assessed on the basis of compressive strength tests on cubic specimens and tensile strength in beam bending tests, carried out in accordance with standards. The results presented confirm that the use of foamed geopolymer aggregates enables a simultaneous increase in thermal insulation and the design of ultra-lightweight structural elements with sufficient load-bearing capacity for chimney systems (including suspended ones). This combination of low thermal conductivity, reduced mass, and appropriate mechanical properties makes geopolymer concretes with lightweight mineral and geopolymer aggregates a promising alternative to traditional ceramic materials.

## 1. Introduction

The development of lightweight, energy-efficient, and chemically resistant building materials is becoming one of the key challenges in modern construction [1,2,3]. Chimney components belong to a particularly demanding group of products, as during operation they are exposed to elevated temperatures, cyclic thermal loads, moisture, aggressive flue gas condensates, and mechanical loads resulting from their own weight and installation method [4,5,6]. Traditional ceramic liners and components based on expanded clay concrete, despite their widespread use, are characterized by relatively high weight, limited thermal insulation, and susceptibility to chemical degradation, which necessitates the use of additional insulation layers and increases the complexity of entire chimney systems [7,8].

In recent years, alkali-activated materials and geopolymers have been gaining increasing attention as alternatives to traditional cementitious materials, particularly in high-temperature and chemically aggressive applications [9,10,11,12]. Geopolymers are characterized by high resistance to acids, low shrinkage, good thermal stability, and a significantly lower carbon footprint compared to Portland cement [13,14]. These properties make them theoretically particularly attractive for use in flue gas system components, chimney liners, and fire-resistant enclosures [15,16]. Currently, geopolymer materials are often produced using metallurgical waste [17,18,19]. Recent studies on geopolymer materials confirm their growing importance as sustainable binding materials based on industrial waste. Li et al. developed eco-friendly injection materials based on geopolymers with the addition of red mud, ash, and calcium oxide, exhibiting adequate fluidity, setting time, and high mechanical strength (up to approx. 57.7 MPa) while incorporating an increased proportion of steel mill waste, indicating the potential for practical application of these materials in underground engineering and soil waterproofing [20]. In contrast, the review by Gou et al. focuses on cementitious and geopolymer composites containing lithium slag, summarizing the current state of knowledge regarding the modification of binding matrices using this metallurgical waste. The authors emphasize that the incorporation of lithium slag can improve the mechanical properties, rheology, and microstructure of both multi-component concretes and geopolymers, which opens new prospects for increasing the durability and performance of construction materials in the context of the circular economy [21]. Similarly, increasing attention has been paid to the use of red mud (bauxite residue) as a precursor or additive in alkali-activated materials and geopolymers. Red mud, generated in large quantities by the alumina industry, is rich in aluminosilicate and iron oxide phases, making it a promising candidate for geopolymer synthesis. Its incorporation into cementitious and geopolymer systems may contribute to reducing CO_2_ emissions and limiting the consumption of primary raw materials, in line with circular economy principles. At the same time, challenges such as high alkalinity, variability in composition, and the presence of trace metals require appropriate pretreatment and stabilization methods. Nevertheless, with proper processing, red mud can be successfully utilized in geopolymers and other construction materials, offering a viable pathway for the valorization of industrial waste and the development of low-carbon binders [22]. Furthermore, recent studies have demonstrated that the performance of red mud in geopolymer systems can be significantly enhanced through activation techniques, such as alkali–thermal treatment. Such approaches increase the reactivity of red mud, enabling the development of high-performance geopolymers with improved mechanical properties and reduced environmental impact. These findings confirm that appropriately processed red mud can serve not only as a supplementary material but also as an effective precursor in advanced geopolymer systems [23].

One of the current trends in the development of geopolymer concretes is their design as lightweight composites through the use of porous mineral or geopolymer aggregates, which allows for a simultaneous reduction in bulk density and thermal conductivity while maintaining acceptable mechanical properties [24,25,26,27]. Studies indicate that lightweight aggregates, such as expanded clay, perlite, or foamed inorganic materials, can significantly improve the thermal insulation properties of geopolymer concretes; however, their introduction is associated with marked changes in the microstructure of the aggregate–matrix interface, which directly affects the load-bearing capacity and failure mechanism of the composite [28,29,30]. In particular, the use of foamed geopolymer aggregates, manufactured from the same class of materials as the binding matrix, is viewed as a solution that improves the chemical and thermal compatibility of the composite’s components and reduces structural degradation under elevated temperature conditions [27,31,32]. Despite the growing number of publications on lightweight geopolymer concretes, there is still a lack of comprehensive studies on their mechanical and functional behavior in applications specific to chimney systems, where the simultaneous effects of temperature, moisture, aggressive condensates, and installation loads—including in solutions with suspended elements—are of critical importance [5,33,34].

One of the main challenges in designing geopolymer concretes for chimney applications is meeting several conflicting requirements. Low thermal conductivity is key, as it helps reduce heat loss and lower the temperature of the chimney’s outer walls. Low material density is also important, as it enables the design of lightweight chimney systems, including suspended ones. At the same time, it is necessary to maintain sufficient mechanical strength to ensure safe installation and reliable operation of the components under service conditions. Previous studies have shown that the use of lightweight mineral aggregates, such as expanded clay and perlite, significantly reduces the density and thermal conductivity of geopolymer concretes; however, this often comes at the expense of mechanical strength [35]. In a previous study, the authors presented a detailed analysis of the thermal properties of geopolymer concretes with lightweight aggregates, highlighting their high potential for use in chimney system components [35]. However, there was a lack of in-depth analysis of their mechanical behavior, which in practice determines the feasibility of using these materials in actual structures. A promising approach to overcoming the trade-off between low density and strength is the use of aggregates made of foamed geopolymers. Unlike conventional lightweight aggregates, foamed geopolymer granules are chemically and structurally compatible with the geopolymer matrix, which promotes better bonding at the phase interface and more efficient stress transfer. At the same time, their highly porous internal structure ensures very low thermal conductivity, allowing them to serve simultaneously as a lightweight filler and a thermal insulation layer.

**Figure 1:**
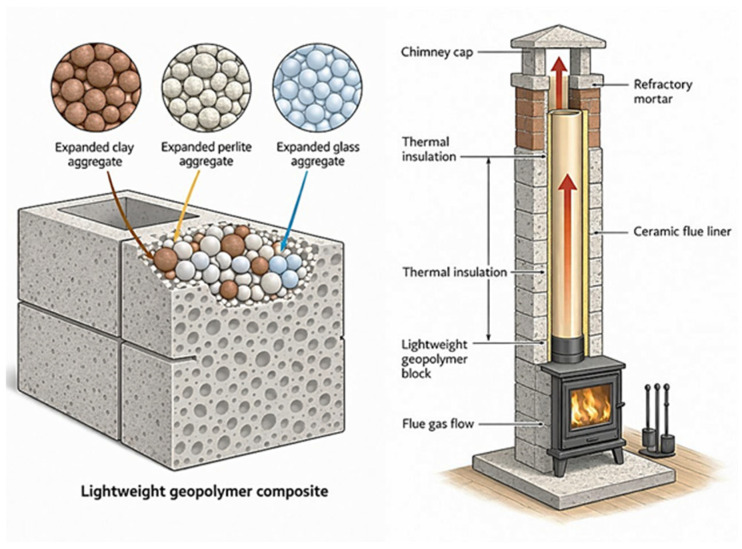
Schematic diagram illustrating the use of geopolymer concrete with lightweight aggregates in the construction of chimney flues (an image generated using artificial intelligence–ChatGPT, GPT-5.3 model, OpenAI).

This article addresses an identified research gap and presents a comprehensive analysis of the properties of geopolymer concretes modified with foamed geopolymer aggregates and lightweight mineral aggregates, such as expanded clay and perlite. The scope of the research includes, in particular, the evaluation of mechanical parameters, including compressive strength and tensile strength in a flexural test, which are key design criteria for chimney flue components. Concurrently, the thermal properties of geopolymer concretes with lightweight aggregates were analyzed, with particular emphasis on their thermal conductivity and potential for use in chimney flues. A comparison of the mechanical and thermal test results enabled the formulation of design principles for lightweight geopolymer concretes capable of simultaneously bearing loads and reducing heat loss in modern chimney systems. As a result, the proposed solutions can serve as a durable, energy-efficient, and low-emission alternative to traditional ceramic liners and expanded clay concrete-based components. A schematic diagram of the application of geopolymer concretes with lightweight aggregates in chimney flue construction is presented below in Figure 1.

The research presented in this article represents a significant and innovative contribution to advancing scientific knowledge in the field of geopolymer materials, particularly in the design of lightweight composites that combine high thermal insulation with the required mechanical properties. The results obtained expand the current state of knowledge regarding the influence of the type, structure, and properties of lightweight geopolymer and mineral aggregates on the mechanical behavior of geopolymer concretes. Furthermore, this research provides new insights into the potential practical application of such materials in specialized components of building systems, such as chimney flue elements, operating under conditions of elevated temperatures, variable thermal loads, and aggressive chemical environments. The identified relationships between thermal and strength parameters can serve as a basis for further research and development, as well as for the development of design guidelines that promote the implementation of low-emission and sustainable material solutions in modern construction.

The novelty of this study lies in its comprehensive approach, which encompasses both the development of a manufacturing technology for lightweight geopolymer aggregates and their practical application in specialized concretes intended for the production of lightweight chimney liners. Unlike previous studies, which have focused mainly on the synthesis process of geopolymer materials or their general properties, this work proposes an integrated material solution tailored to a specific industrial application. A key novelty is the use of foamed geopolymer mixtures to produce a lightweight, porous aggregate with a controlled structure and physicochemical properties. The use of hydrogen peroxide as a foaming agent, combined with a carefully selected composition of precursors, has made it possible to obtain an aggregate with low density and, at the same time, sufficient mechanical strength. In addition, the developed technological process—which includes controlled curing, crushing, and particle size classification—enables the production of a material with consistent properties, which is crucial for engineering applications. Another innovative aspect of the work is the implementation of the resulting geopolymer aggregate in concrete composites designed for lightweight chimney liners. The proposed solution combines the advantages of geopolymer materials, such as high thermal and chemical resistance, with low thermal conductivity and reduced bulk density. As a result, a structural-insulation material was obtained that can serve as an alternative to traditional solutions used in chimney systems, particularly in the context of improving energy efficiency and service life. Consequently, this work makes a significant contribution to the development of lightweight geopolymer materials and their applications in modern, sustainable construction.

## 2. Materials and Methods

### 2.1. Materials for the Manufacture of Geopolymer Concretes and Foamed Geopolymer Aggregates

The geopolymer concretes and lightweight foamed aggregates were produced using Class F fly ash obtained from the combustion of hard coal at the Skawina Combined Heat and Power Plant (CEZ Skawina S.A., Skawina, Poland), which served as the main precursor in the geopolymerization process. Quartz sand from the Świętochłowice Sand Pit (Świętochłowice, Poland) was used as the mineral filler. Lightweight foamed aggregates were also produced using metakaolin from the Czech Republic (Keramost, Most, Czech Republic). Table 1 presents the chemical composition of the materials used for the production of concrete and lightweight aggregates, while Table 2 shows the particle size distribution.

All mixture variants contained 3.5% by weight of perlite supplied by JAWAR (Glinojeck, Poland). The perlite used is characterized by beneficial physical properties that make it widely applicable in lightweight and thermal insulation materials. This material is white in color and has a refractive index of approximately 1.5, which is typical for amorphous siliceous materials. The pH of the perlite ranges from 6.5 to 8.0, indicating its neutral or slightly alkaline chemical nature. The material’s moisture content is very low, at approximately 0.5%, which contributes to the stability of its physical properties. The specific gravity of perlite ranges from 2.2 to 2.4, while in its expanded form, its bulk density ranges from 60 to 160 kg/m^3^, which indicates its highly porous structure. The particle size of the material ranges from 0 to 2.5 mm, which allows for its easy use in composites and mortars. Perlite exhibits high thermal resistance; its softening point ranges from 871 to 1093 °C, while its melting point ranges from 1260 to 1343 °C. The specific heat of the material is approximately 837 J/kg·K, and its thermal conductivity (λ) ranges from 0.045 to 0.065 W/m·K, making it an effective thermal insulator. Chemically, perlite dissolves in alkalis and hydrofluoric acid, moderately in 1N NaOH solution (less than 10%), slightly in mineral acids (less than 3%), and very slightly in water and weak acids (less than 1%). Thanks to these properties, perlite is a chemically stable material in most operating environments.

To activate the alkaline mixtures and lightweight foamed aggregates, a 10 M aqueous sodium hydroxide solution and an aqueous sodium silicate solution (R-145 sodium silicate) were used in a weight ratio of 1:2.5. The sodium hydroxide solution was prepared from technical NaOH in flake form with a purity of over 99%, supplied by PCC Rokita S.A. (Brzeg Dolny, Poland). The second component of the activator was an aqueous sodium silicate solution with the trade name R-145, characterized by a SiO_2_/Na_2_O molar ratio of 2.5 and a density of approximately 1.45 g/cm^3^, obtained from Zakłady Chemiczne ANSER (Wiskitki, Poland).

**Figure 2:**
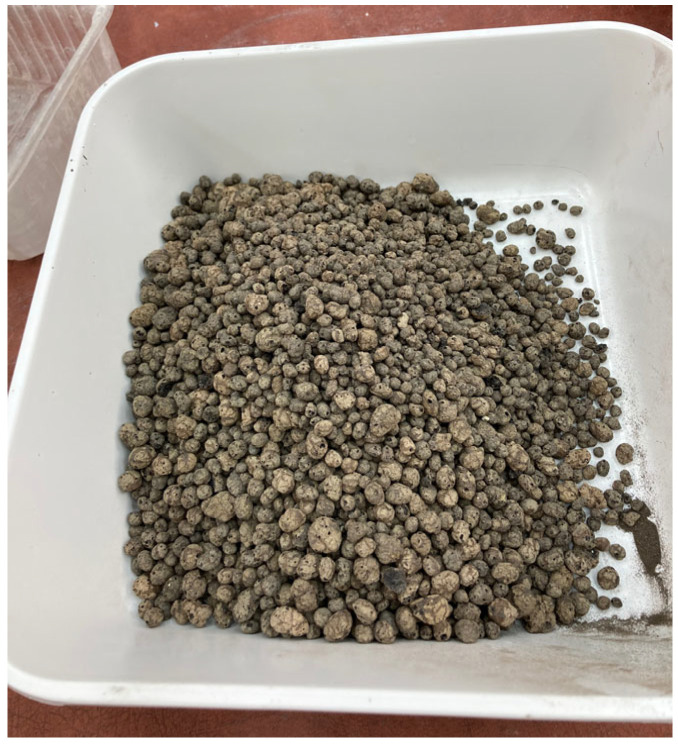
Expanded clay is used as a lightweight aggregate.

Lightweight aggregates were used in the geopolymer concretes, including LECA expanded clay aggregate (KER) produced in Gniew (Poland), fly ash-based foamed geopolymer aggregate (GAFA), and metakaolin-based geopolymer aggregate (GAM). The LECA expanded clay aggregate used, with a particle size of 8–20 mm, is a lightweight material that meets the requirements of EN 15732 [36] and EN 13055-1 [37], confirming its suitability for construction applications. This aggregate is characterized by grains with a nearly spherical shape, which positively affects its rheological properties and compaction in mixtures. The bulk density in a loose state ranges from 246 to 333 kg/m^3^, with an average of approximately 290 kg/m^3^, indicating a low bulk density due to its porous internal structure. The material exhibits a crushing strength of at least 0.75 N/mm^2^, which ensures adequate load-bearing capacity in structural and insulation applications. The thermal conductivity coefficient ranges from 0.095 to 0.160 W/m·K, which confirms its thermal insulation properties, although it is lower than that of materials with more developed open porosity. LECA expanded clay aggregate is also a completely non-combustible material, classified in fire reaction class A1, which makes it safe for applications requiring high fire resistance. Figure 2 shows the expanded clay aggregate used. The fly ash and metakaolin parameters are presented in Table 1 and Table 2 above.

**Figure 3:**
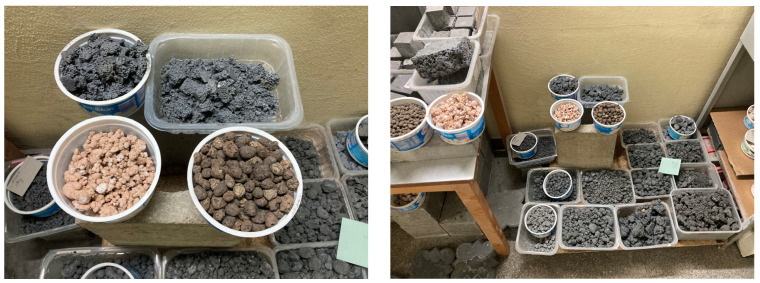
Geopolymer granules made from fly ash and metakaolin, produced by casting slabs and controlled crushing into specific fractions.

To stabilize the porous structure of foamed geopolymer aggregates and increase their mechanical strength, a hydraulic additive in the form of Górkal 70 high-alumina cement, manufactured by Górka Cement Sp. z o.o. was used (Trzebinia, Poland). The foaming process of the geopolymer aggregates was carried out by adding 2.0% by weight of 36% hydrogen peroxide (H_2_O_2_) sourced from Grupa Azoty–Zakłady Azotowe Puławy (Puławy, Poland). Based on previous in-house research and literature reports by other authors, the composition of the mixture intended for the production of geopolymer aggregates was developed. Fly ash or metakaolin was used as the base material in an amount of 38% by weight, while quartz sand, constituting 58% of the mass composition, served as the mineral filler. To stabilize the system, GÓRKAL 70 cement was added at 2% by weight, and 36% hydrogen peroxide (H_2_O_2_) was used as a foaming agent, also at 2% by weight. An alkaline solution was introduced into the mixture prepared in this manner, with the amount selected so that the liquid-to-solid ratio was 0.4:1. The curing process for the foamed geopolymer mixtures was conducted under controlled thermal conditions at a temperature of 75 °C. The time required to achieve sufficient hardness to allow for demolding of the elements was 24 h. The material was molded into specimens measuring 20 × 20 × 5 cm, which were then subjected to controlled crushing and screening to obtain the desired grain size fraction. Geopolymer aggregate with a 5–10 mm fraction was used in the tests. Preliminary crushing was performed using a laboratory jaw crusher (a prototype device developed and manufactured at the Cracow University of Technology, Kraków, Poland), and grain sorting was carried out on an ANALYSETTE 3 PRO laboratory sieve (MERAZET S.A., Poznań, Poland). After a curing period of 28 days, the lightweight aggregates were used to prepare concrete mixtures. The method of producing foamed geopolymer aggregate and its application in lightweight composites have been described in detail in a previous study by the authors [35]. In that work, the influence of material structure and porosity on the properties of the resulting composites was analyzed, highlighting the key role of microstructure in determining mechanical and functional performance. However, detailed characterization of the aggregate at the particle level, including shape and surface-related parameters after crushing, was not the focus of that study. It should be noted that this does not affect the interpretation of the results presented herein, as the primary objective of this work was to evaluate the properties of the composites, while the aggregate was produced using a consistent and repeatable procedure. Sample photographs of selected materials, illustrating the produced aggregates, are presented in the figure labeled as Figure 3.

The components of the individual mix variants and their designations are summarized in Table 3. The mixed compositions, expressed per 1 m^3^, were determined using the volumetric method based on the laboratory mix design and the assumed bulk densities of the components. The calculations are theoretical and do not account for compaction effects, void filling, or changes in the mixture’s volume after mixing. The sample names presented in Table 3 are based on the type of lightweight aggregate used and the amount of alkaline activator solution used in a given mixture.

### 2.2. Manufacturing of Geopolymer Concretes Using Lightweight Aggregates

The geopolymer concretes were produced using a GEOLAB (Warsaw, Poland) M/LMB-s laboratory mortar and concrete mixer. The mixture preparation process began with the preliminary mixing of dry ingredients, including Class F fly ash and quartz sand, for approximately five minutes at the mixer’s maximum operating speed of 100 rpm. After the dry mixing stage was completed, a previously prepared alkaline activating solution, consisting of an aqueous solution of NaOH and sodium silicate, was gradually introduced into the system according to the specified weight ratio. The addition of the activator initiated the next mixing stage, lasting approximately three minutes, until a uniform and homogeneous consistency of the geopolymer mixture was achieved. In the next stage, components intended to reduce the concrete’s bulk density and improve its thermal insulation properties were added to the fresh mixture, including lightweight aggregates such as foamed geopolymer aggregates and expanded clay, as well as foamed perlite serving as a lightweight filler. After adding them, mixing continued for another five minutes at a reduced mixer speed of approximately 50 rpm, which allowed for uniform distribution of the ingredients while minimizing damage to the porous structure of the aggregates. The resulting concrete mixture was then placed in molds and subjected to de-airing and preliminary compaction on a laboratory vibrating table for approximately three minutes to remove excess air and achieve the appropriate degree of compaction. After forming, the specimens were placed in a laboratory dryer (POL-EKO Perfect-Environment, Wodzisław Śląski, Poland) and subjected to initial curing at 75 °C for 24 h, which was intended to accelerate the polycondensation process and stabilize the geopolymer matrix. After completing this stage, the samples were removed from the molds and cured for a period of 28 days prior to further testing.

### 2.3. Testing of Thermal Conductivity Coefficient of Geopolymer Concretes Using Lightweight Aggregates

**Figure 4:**
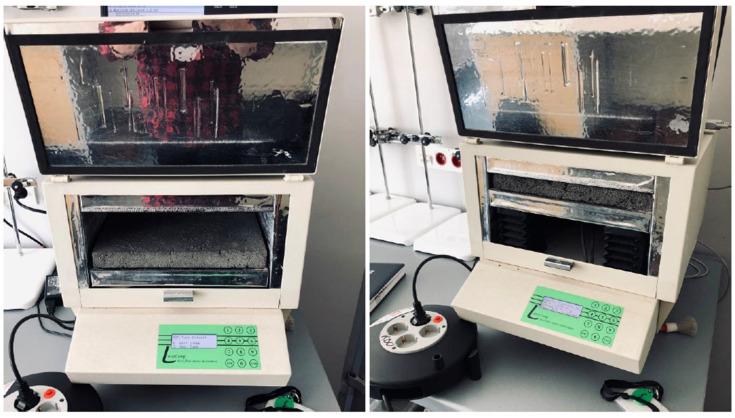
The test sample was placed in the plate apparatus.

Tests of the thermal conductivity (λ) of the foamed geopolymer were conducted on 36 plate-shaped samples measuring 300 × 300 × 30 mm. Four tests were performed for each mixture, and the average value was calculated. The measurements were performed using the FOX314 Software (WinTherm32); (TA Instruments: New Castle, DE, USA) thermal conductivity tester, equipped with dedicated WinTherm32 software. The device has a measurement range of 0.005 to 0.35 W/(m·K), with an expanded uncertainty of 1%, and complies with the requirements of ASTM C518 [38] and ISO 8301 [39]. The test procedure was carried out in accordance with the manufacturer’s guidelines and instructions. For the measurements, a temperature difference of 30 °C between the device’s heating and cooling plates was assumed. The temperature of the cooling plate was set at 0 °C, while the temperature of the heating plate was 30 °C, which corresponded to a system average temperature of 15 °C. Measurements were conducted until a steady-state heat flux through the sample was reached, which was automatically monitored and signaled by the apparatus control software. Figure 4 shows an example of a test sample placed in the plate apparatus during the measurement.

### 2.4. Testing of Mechanical Properties of Geopolymer Concretes Using Lightweight Aggregates

Compressive strength tests on geopolymer and concrete specimens were conducted on cubic specimens in accordance with the requirements of PN-EN 206 206+A2:2021-08 [40] and PN-EN 12390-3:2019-07 [41]. A total of 90 specimens measuring 100 × 100 × 100 mm were tested. Ten measurements were taken for each specimen, and the average value was calculated. The tests were performed using an Advantest 9 universal testing machine (CONTROLS Polska Sp. z o.o., Warsaw, Poland), characterized by a measurement range from 15 to 3000 kN, with an expanded uncertainty of 0.25% for the 600 kN range. The test procedure was conducted in accordance with standard guidelines, assuming a constant stress rate of 0.5 MPa/s. According to the standard, the stress rate may range from 0.2 to 1.0 MPa/s. The load was applied perpendicular to the direction of specimen forming, which was intended to ensure the comparability of results and to reflect the actual operating conditions of the material. Compression tests were conducted until the specimens were destroyed, and the obtained values of maximum stresses were used to determine the compressive strength of the tested materials.

**Figure 5:**
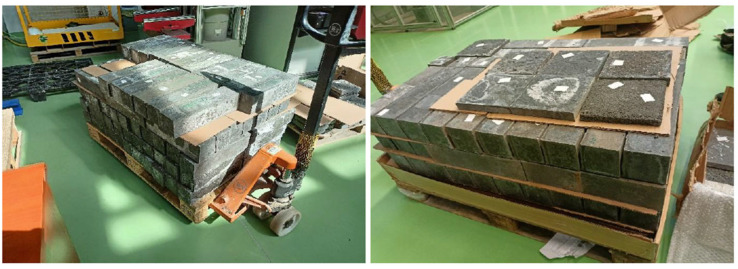
Specimens were prepared for testing tensile strength under bending and compression.

Tensile strength tests on geopolymer and concrete beams were carried out in accordance with the requirements of PN-EN 206 206+A2:2021-08 [40] and PN-EN 12390-5:2019-08 [42]. A total of 90 specimens measuring 100 × 100 × 400 mm were tested. Each specimen was tested 10 times, and the average value was then calculated. An MTS 319.25 testing machine (MTS System Corp., Eden Prairie, MN, USA), with a measurement range of 5 to 250 kN and an expanded uncertainty of 0.25% for the 10 kN range. The tests were performed using the three-point bending method, in accordance with the diagram presented in the annex to the PN-EN 12390-5:2019 standard. In accordance with the standard guidelines, a constant stress rate of 0.05 MPa/s (as specified in the standard, ranging from 0.2 to 1.0 MPa/s) and a lower support spacing l of 300 mm were adopted. The load was applied perpendicular to the direction of specimen formation, which ensured the repeatability and comparability of the results obtained. The tests were conducted until the beams failed, with the fracture occurring in all cases in the area between the lower supports, in accordance with the requirements of the PN-EN 12390-5:2019 standard. Figure 5 shows the specimens prepared for compressive strength and tensile strength tests in the bending test.

### 2.5. Additional Measuring Devices Used in the Tests

During the experimental work, additional measuring equipment was used, including an electronic caliper with a measuring length of 500 mm, a range of 0–500 mm, and a resolution of 0.01 mm, as well as a RadWag WLC 30/C1/R laboratory scale (Radwag, Radom, Poland), with a measurement range of 0–30 kg, which were used to precisely determine the geometric dimensions and mass of the tested samples. Furthermore, to verify and continuously monitor laboratory conditions, a TFA Dostmann H560 Dewpoint Pro digital thermohygrometer (No. 3–15-WW) (TFA Dostmann GmbH & Co. KG, Wertheim, Germany) was used to verify and monitor laboratory conditions in real time; this device enables the measurement of temperature in the range of −40 to 70 °C (resolution 0.1 °C, uncertainty ±0.5 °C) and relative humidity in the range of 0–99% RH (resolution 0.1%, uncertainty ±3%). Due to the large number of samples tested and the time-consuming nature of the individual testing procedures, environmental parameters were systematically monitored throughout the testing period. This allowed for the confirmation of the stability of temperature and humidity conditions and ensured the comparability and reliability of the obtained results. All of the tests described above were conducted at the Construction Laboratory of the Faculty of Civil Engineering and Architecture at the Lublin University of Technology.

### 2.6. AI-Assisted Analysis

Selected sections of the manuscript, particularly Section 3 and Section 4, were linguistically enhanced and stylistically refined using the artificial intelligence tool ChatGPT (OpenAI, model GPT-5.3, 2026 version). The AI tool was employed to support the authors in improving clarity, coherence, and language fluency, while fully preserving the scientific integrity and authorship of the work. Additionally, Figure 1 and Graphical Abstract was prepared with the assistance of artificial intelligence using ChatGPT (GPT-5.3, OpenAI) to enhance its visual quality and clarity, while maintaining consistency with the scientific content presented in the manuscript.

## 3. Results

### 3.1. Thermal Conductivity Coefficient of Geopolymer Concretes Using Lightweight Aggregates

**Figure 6:**
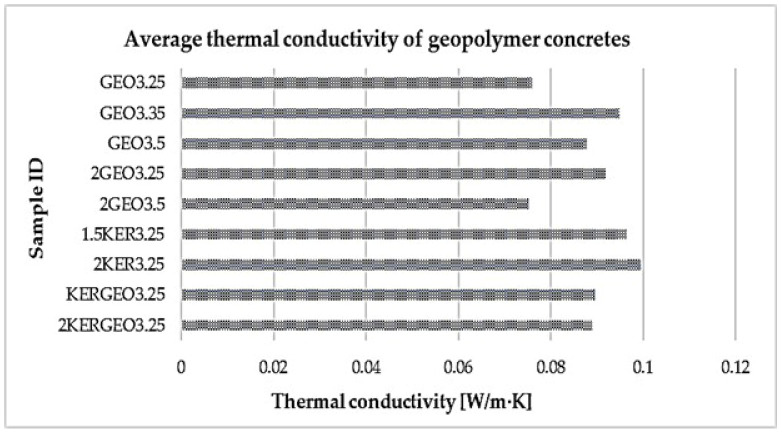
Thermal conductivity of geopolymer concretes with lightweight aggregates.

Table 4 presents the average thermal conductivity values obtained for the analyzed materials, while Figure 6 presents the same results in graphical form, illustrating the variation in thermal conductivity of geopolymer concretes with lightweight aggregates. This combination of tabular and graphical data allows for a clear comparison of the influence of the type and characteristics of the aggregate used on the thermal properties of geopolymer composites. The obtained results also enable an assessment of the insulating potential of the tested concretes in the context of their possible application in chimney system components, where low thermal conductivity is one of the key performance parameters.

An analysis of the results presented in Table 4 and Figure 6 clearly confirms the significant influence of both the amount of alkaline activator solution and the type of lightweight aggregate used on the thermal conductivity of geopolymer concretes. The lowest λ values, at approximately 0.075 W/(m·K), were obtained for the GEO3.25 and 2GEO3.5 samples, indicating the very high insulating potential of these materials. The values obtained are comparable to the parameters characteristic of foamed geopolymers and lightweight insulating concretes described in the literature, in which a high content of closed pores and limited continuity of the solid phase play a decisive role [43,44,45]. Increasing the amount of alkaline activator solution, as seen, for example, in the case of sample GEO3.35, results in an increase in the λ coefficient to values close to 0.095 W/(m·K). This phenomenon can be attributed to the intensification of the geopolymerization process, leading to the densification of the matrix microstructure and a reduction in the proportion of large pores, which promotes more efficient heat conduction [46]. Similar relationships between the degree of structural densification and the increase in thermal conductivity were observed in studies of lightweight geopolymers based on fly ash [47,48]. The introduction of expanded clay as a lightweight aggregate results in a further increase in the λ value to the range of 0.096–0.100 W/(m·K), which is due to the higher thermal conductivity of the mineral aggregate itself and the more continuous solid-phase matrix compared to foamed geopolymer aggregates. Hybrid systems combining expanded clay and geopolymer aggregates exhibit intermediate λ values, at around 0.089 W/(m·K), which confirms the possibility of deliberately shaping thermal properties through the selection of aggregate composition [49]. From the perspective of applications in chimney systems, these results indicate the high potential of geopolymer concretes as materials that simultaneously serve structural and thermal insulation functions.

### 3.2. Mechanical Properties of Geopolymer Concretes Using Lightweight Aggregates

**Figure 7:**
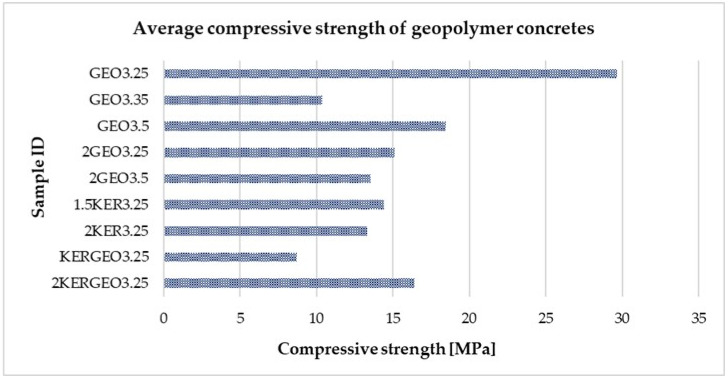
Compressive strength of the tested geopolymer concretes with lightweight aggregates.

Table 5 below summarizes the average compressive strengths obtained for the tested geopolymer concretes, while Figure 7 presents the same results graphically. This combination of tabular and graphical data allows for a clear analysis of the influence of mix composition, particularly the type and proportion of lightweight aggregates, on the materials’ ability to withstand compressive loads. The obtained results allow for an assessment of the suitability of the developed geopolymer concretes for use in chimney flue components, where adequate mechanical load-bearing capacity is required while simultaneously reducing self-weight and improving thermal insulation properties.

The compressive strengths obtained fall within the range typical for lightweight concrete and geopolymer concrete with porous aggregates. In the GEO series, a systematic increase in strength is observed with an increase in the amount of activator solution, from 5.96 MPa (GEO3.25) to 18.47 MPa (GEO3.5), which indicates a significant influence of the degree of matrix compaction on the material’s load-bearing capacity [24,50]. Increasing the proportion of lightweight aggregate results in moderate strength values ranging from 13.55 to 15.08 MPa, confirming the trade-off typical of lightweight concretes between reduced density and mechanical properties [24,51]. The use of expanded clay results in stable strength values in the range of 13.30–14.41 MPa, which is consistent with the beneficial effect of porous mineral aggregates on the structural homogeneity and repeatability of the mechanical behavior of lightweight concrete observed in the literature [51,52]. The most balanced mechanical properties among the mixed systems were obtained for the 2KERGEO3.25 variant (16.38 MPa), confirming that hybrid systems are an effective way to combine acceptable load-bearing capacity with a reduction in the material’s self-weight [24,52]. High standard deviations may result from the use of aggregates with different parameters and from the fact that different liquid contents are used, which could lead to inhomogeneities.

**Figure 8:**
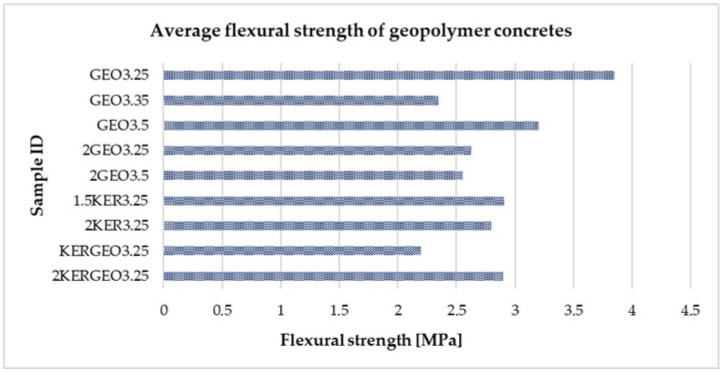
Flexural tensile strength of the tested geopolymer concretes with lightweight aggregates.

Table 6 below summarizes the results of the flexural tensile strength tests for the geopolymer concretes under study, while Figure 8 presents the same data in graphical form. This presentation of the results allows for a clear analysis of the influence of the type of lightweight aggregate used and the mix composition on the material’s ability to withstand tensile stresses arising under bending conditions. The presented results are of significant importance for the design of chimney duct components, which during installation and operation are exposed to bending loads resulting from both the components’ own weight and thermal and installation-related effects.

The average flexural tensile strengths of the tested geopolymer concretes range from 2.20 MPa to 3.85 MPa, which is typical for lightweight concretes and concretes with porous aggregates. The highest value was obtained for sample GEO3.25 (3.85 MPa), indicating that despite its low compressive strength, this material exhibits good flexural resistance, typical of composites with high porosity and an increased ability to redistribute tensile stresses [53,54]. Increasing the amount of activator solution in the GEO series leads to a decrease in strength to the range of 2.35–3.20 MPa and to an increase in the variation of results, suggesting a shift toward a more brittle failure mechanism as the matrix becomes denser. Similar trends are described in recent studies on lightweight concretes, in which an increase in matrix stiffness causes a decrease in flexural strength [54,55]. Variants with an increased proportion of lightweight aggregate and with the addition of expanded clay (2GEO3.25, 2GEO3.5, 1.5KER3.25, 2KER3.25) exhibit stable values in the range of 2.55–2.91 MPa with low standard deviations, indicating more uniform material behavior under bending conditions. The most balanced properties were obtained for the 2KERGEO3.25 hybrid variant (2.90 MPa), confirming that combining different types of lightweight aggregates promotes the stabilization of flexural strength, in line with trends observed in the literature on lightweight concrete [55,56].

**Figure 9:**
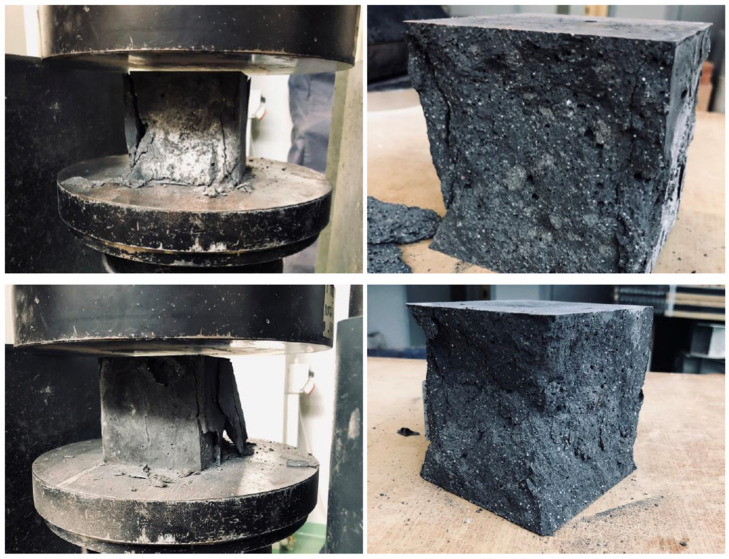
Examples of damage to samples in a compression test.

Figure 9 below shows examples of failure patterns observed in the specimens during compressive strength testing. Analysis of the failure modes and mechanisms revealed that all tested specimens failed in a normal manner, in accordance with the guidelines of the PN-EN 12390-3:2019-07 standard [41]. The observed cracks and fractures most often initiated in the central part of the specimens and propagated toward the side surfaces, leading to characteristic splitting or crushing of the material under the influence of increasing compressive load. No undesirable forms of failure were observed, such as slippage at the contact surfaces with the press plates, premature chipping of corners, or damage resulting from improper axial loading. This indicates that the sample preparation process, the method of positioning the samples in the testing machine, and the test conditions were correct and did not negatively affect the results obtained. The observed failure patterns also confirm the uniform behavior of the material under compression and the reliability of the strength values obtained.

**Figure 10:**
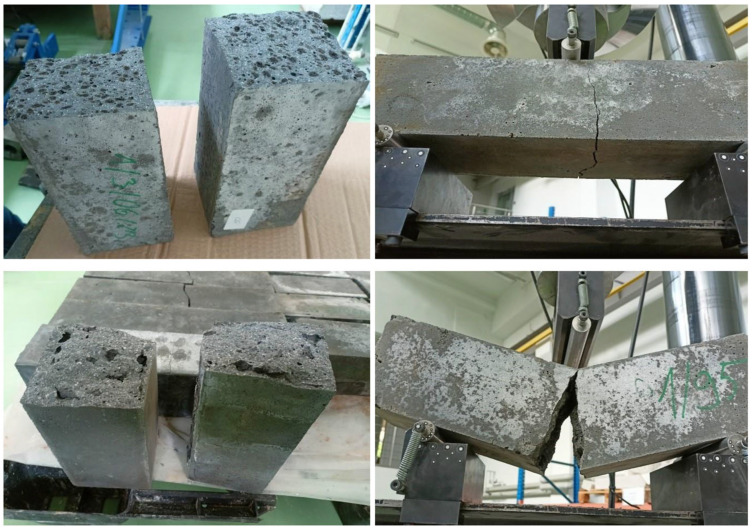
Examples of damage to samples in a bending test.

Figure 10 below shows examples of fracture patterns in the specimens obtained during the bending tensile strength tests. Analysis of the crack propagation showed that all tested specimens failed in the area between the lower supports, which is consistent with the guidelines of the PN-EN 12390-5:2019 standard. Failure occurred in the zone of maximum tensile stresses, located in the lower part of the specimen cross-section, and the cracks were single or dominant in nature, typical of a properly conducted three-point bending test. No abnormal failure modes were observed, such as cracks initiating in the support region, specimen slippage, local crushing at points of contact with the loading beam, or damage resulting from improper specimen positioning. This indicates that the test conditions, specimen geometry, and loading method ensured the correct conduct of the test and the reliability of the results obtained. The observed fracture patterns confirm the homogeneous behavior of the material under bending conditions and the accurate representation of the failure mechanism characteristic of lightweight and geopolymer concretes.

### 3.3. Selection of the Optimal Solution of Geopolymer Concretes Using Lightweight Aggregates

**Figure 11:**
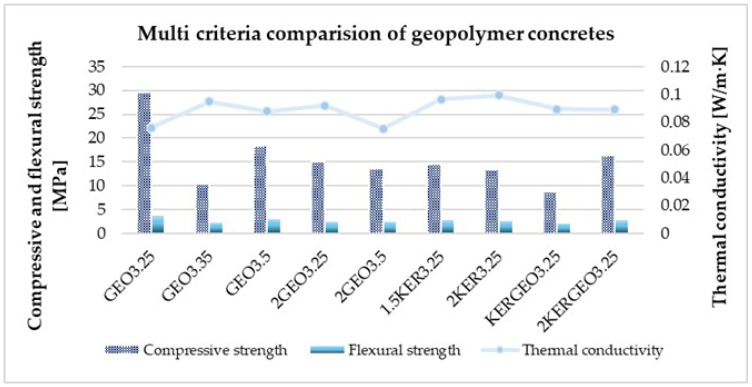
Multi-criteria chart of the properties of the tested geopolymer concretes with lightweight aggregates.

To enable the unambiguous identification of optimal mixtures, a multi-criteria diagram was developed that provides a concise overview of the balance among the analyzed solutions in terms of key performance properties (Figure 11). The analysis simultaneously considered three independent metrics describing material properties relevant to their potential applications. This approach allows for an assessment of the trade-off between individual material properties and the identification of mixtures characterized by the most balanced set of parameters, rather than maximizing just one of them. The use of a composite index also facilitates the comparison of variants with different compositions and identifies solutions that best combine requirements for mechanical load-bearing capacity and thermal insulation properties. Consequently, the multi-criteria chart serves as a useful tool to support the design process of lightweight geopolymer concretes that serve both structural and insulating functions.

The high ranking of the GEO3.25 mixture is primarily due to its very good thermal insulation properties and high flexural strength, despite its limited compressive strength. This type of behavior is characteristic of lightweight concretes with high porosity, in which the ability to redistribute tensile stresses can compensate for reduced compressive strength, as is widely described in the literature on concretes with porous aggregates [57,58]. The GEO3.5 and 2GEO3.5 mixtures are characterized by a more uniform distribution of all three analyzed metrics, accompanied by increased compressive strength. This balance of properties confirms the trend observed in studies on lightweight concretes, according to which an increase in the degree of matrix compaction improves mechanical load-bearing capacity at the expense of a partial deterioration in thermal insulation; however, with an appropriate selection of composition, it is possible to maintain a beneficial compromise [54,57]. Mixes containing 1.5KER3.25 and 2KER3.25 expanded clay aggregate exhibit intermediate characteristics, indicating stable but less extreme performance properties. The use of porous mineral aggregate promotes a more homogeneous microstructure and predictable mechanical behavior, which is consistent with the results of studies on lightweight concretes based on expanded clay and perlite [54,59]. Improved mechanical stability does not always go hand in hand with maximized thermal insulation. Hybrid systems, in particular 2KERGEO3.25, confirm the validity of combining mineral and geopolymer aggregates to achieve more balanced performance properties. This approach is increasingly cited in the literature as an effective strategy for designing lightweight concretes with both structural and insulating functions [58,59]. In contrast, the KERGEO3.25 mixture has a lower multi-criteria rating, indicating a less favorable trade-off between thermal and mechanical properties in this variant. A comprehensive analysis of all tested compositions (GEO3.25, GEO3.35, GEO3.5, 2GEO3.25, 2GEO3.5, 1.5KER3.25, 2KER3.25, KERGEO3.25, and 2KERGEO3.25) confirms that the optimal selection of mixtures does not rely on the a single property, but on consciously balancing thermal insulation, compressive strength, and flexural strength, which is consistent with current trends in the design of lightweight concrete and low-density materials [54,57,58,59]. The results clearly indicate that the use of mixtures of lightweight ceramic and geopolymer aggregates allows for the production of geopolymer concretes with balanced mechanical properties, combining reduced density with sufficient load-bearing capacity and good reproducibility of results, which is crucial in the context of precast chimney elements. The obtained values are fully sufficient for prefabricated chimney components, which in reality do not function as primary load-bearing elements but must only support their own weight, installation weight, and local service loads.

### 3.4. Chimney Systems with Geopolymer Concretes Using Lightweight Aggregates

**Figure 12:**
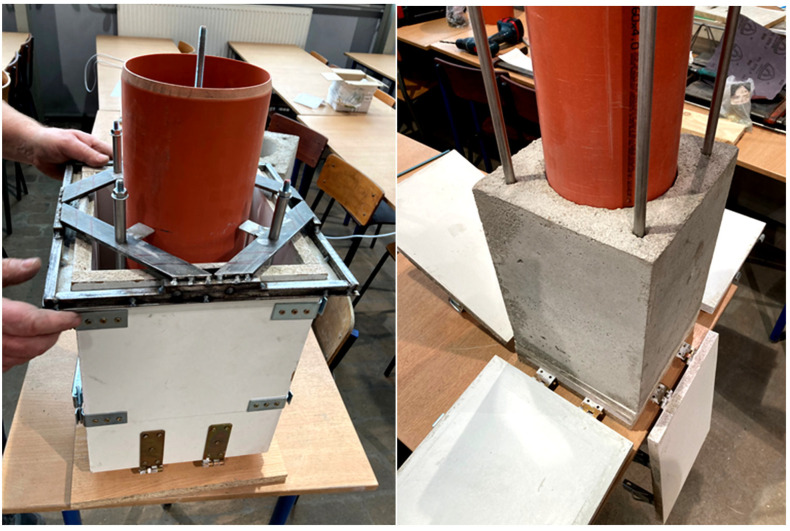
A prototype mold used to manufacture chimney system components from geopolymer materials.

Figure 12 shows a prototype mold used to manufacture chimney system components from geopolymer materials. The designed mold enables the production of components with geometries corresponding to actual chimney flue components, while maintaining the required dimensional accuracy and repeatability of the molding process. The mold’s design allows for proper compaction of the geopolymer mixture, control over the distribution of lightweight aggregates, and the achievement of a homogeneous material structure throughout the entire cross-section of the component. The prototype mold used represents a significant step in the transition from laboratory testing of samples to the production of components on a semi-industrial scale, enabling the assessment of the manufacturability of geopolymer mixtures and their behavior during molding, curing, and demolding. The elements obtained in this way allow for the verification of the practical applicability of the developed geopolymer concretes in chimney systems, particularly in the context of producing lightweight, prefabricated components with both structural and insulating functions.

**Figure 13:**
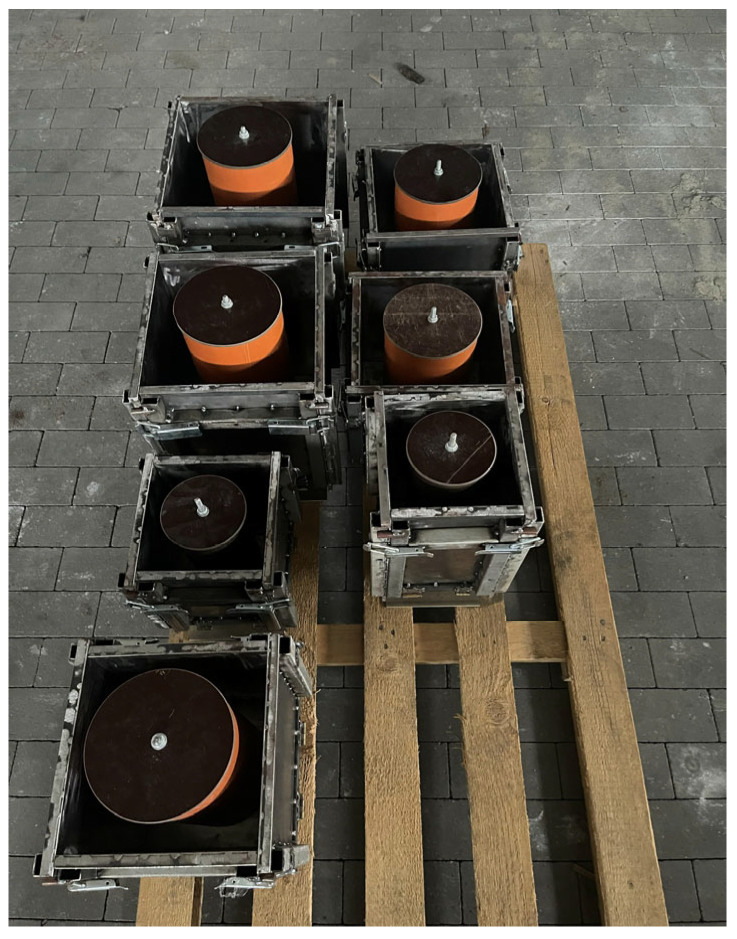
A specialized steel mold for manufacturing chimney system components made of geopolymer concrete.

Figure 13 shows the final steel mold designed for manufacturing chimney system components made of geopolymer concrete. This mold was developed based on experience gained during tests using a prototype and represents the final solution, adapted to semi-technical and industrial production conditions. The use of a steel structure ensures high rigidity and geometric stability of the mold, which allows for the production of components with consistent dimensions, smooth surfaces, and an appropriate finish quality. A properly designed steel mold also allows for repeated use without loss of geometric parameters and enables effective compaction and curing of the geopolymer mixture. Its design facilitates easy demolding of the elements and control of the manufacturing process, which is crucial for the practical implementation of the developed geopolymer materials in the production of chimney system components.

**Figure 14:**
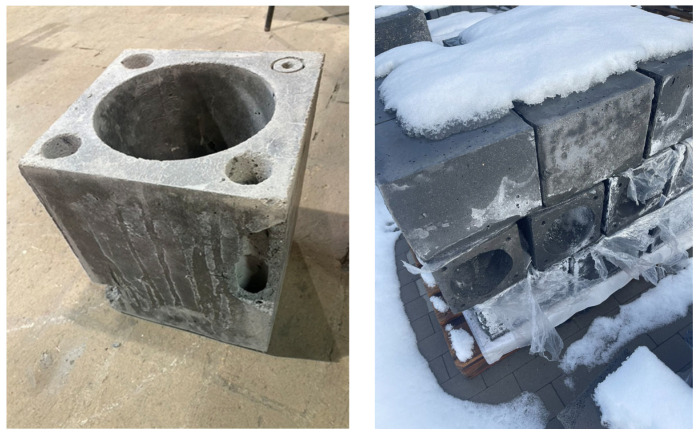
A prototype component made of geopolymer concrete with lightweight aggregates—designed for the construction of a flue.

Figure 14 shows a prototype component made of geopolymer concrete with lightweight aggregates, intended for the construction of a flue gas chimney. The component shown was manufactured using the developed geopolymer mixtures and a steel production mold, which allowed for an assessment of the material’s practical applicability in actual chimney system components. The prototype features regular geometry, adequate surface quality, and a homogeneous material structure, confirming the mixture’s good workability and its ability to properly fill the mold. The use of lightweight aggregates made it possible to obtain an element with reduced self-weight while maintaining the required mechanical and thermal insulation properties. The presented prototype represents a significant stage in the verification of the developed technology and confirms the potential of geopolymer concretes with lightweight aggregates as materials for the production of modern, lightweight, and energy-efficient flue chimney components.

## 4. Discussion

This article presents the results of research on geopolymer concretes based on fly ash, in which both lightweight geopolymer aggregates obtained by foaming and conventional lightweight aggregates, such as expanded clay and perlite, were used as fillers. The research aimed to determine the compressive strength and flexural strength of the composites produced, as well as their thermal conductivity coefficient.

A key aspect influencing the mechanical performance of the developed composites is the nature of the aggregate–matrix interface. In the case of foamed geopolymer aggregates, a higher degree of physicochemical compatibility with the fly ash-based geopolymer matrix can be expected compared to conventional lightweight aggregates such as expanded clay (LECA). Both the aggregate and the matrix are based on aluminosilicate systems activated under alkaline conditions, which promotes the formation of a more continuous interfacial transition zone (ITZ) and improves stress transfer across the interface [27,28]. In contrast, LECA aggregates, despite their porous internal structure, are characterized by a sintered ceramic surface with limited chemical reactivity, resulting in a weaker and more mechanical-type bond with the geopolymer matrix [56,58]. This difference in interfacial behavior may explain the generally more homogeneous mechanical response observed in systems containing geopolymer aggregates. The observed discrepancy between flexural and compressive strength in the GEO3.25 mixture can be attributed to its highly porous structure and lower degree of matrix densification. As shown in the results, this composition exhibits relatively low compressive strength but the highest flexural strength among the tested variants. Such behavior is typical of lightweight composites with a significant proportion of porous aggregates, where the presence of distributed pores and compliant phases allows for more effective stress redistribution under bending [56,58]. Under compressive loading, however, these pores act as stress concentrators, leading to premature failure and reduced strength. Additionally, the lower content of the activating solution in GEO3.25 likely results in a less compact geopolymer matrix, further reducing compressive strength while maintaining a certain level of flexibility and crack-bridging capability under bending conditions. This indicates that, in lightweight geopolymer concretes, the balance between matrix densification and aggregate porosity plays a decisive role in determining the relationship between compressive and flexural strength. Mixtures with lower activator content and higher porosity tend to exhibit more ductile behavior under bending but reduced resistance to compressive loads, whereas increasing matrix compactness enhances compressive strength at the expense of flexural performance [53,56].

Despite numerous reports in the literature on geopolymer concretes, previous studies have focused to a limited extent on analyzing their mechanical properties in the context of their potential use in chimney system components [4,5,6]. In such applications, not only is an adequate level of mechanical strength crucial, but also a low bulk density of the material and favorable thermal insulation properties [5,34]. For this reason, the composites produced contained significant amounts of lightweight aggregates, which had a significant impact on their mechanical strength.

The results obtained indicate that geopolymer concretes with the addition of aggregates in the form of foamed geopolymers, expanded clay, and perlite can meet the requirements for materials used in chimney systems. Additionally, as demonstrated in the authors’ previous work [35], these types of materials are characterized by high resistance to elevated temperatures, reaching up to 800 °C, which significantly exceeds the fire resistance of conventional Portland concretes.

In addition to their use in chimney systems, lightweight geopolymer concretes with the addition of foamed aggregates, expanded clay, and perlite have a wide range of potential applications in many areas of civil and materials engineering. Due to the advantageous combination of low bulk density, high thermal resistance, and good mechanical properties, these materials can be considered as an alternative to traditional lightweight concretes and ceramic materials in structural and cladding elements exposed to elevated temperatures [9,10,57].

Their use in energy and industrial infrastructure elements, such as furnace casings, exhaust ducts, process pipe covers, fire barriers, and insulating structural elements in high-temperature installations, is particularly promising. Thanks to their high structural stability at temperatures of several hundred degrees Celsius, geopolymer lightweight concretes can also be used in specialized construction, including industrial facilities, commercial power engineering, and waste thermal treatment installations [60,61].

Another important area of development is the use of these materials in the prefabrication of building components with increased fire resistance and thermal insulation, such as wall panels, cladding panels, chimney inserts, ceiling elements, and lightweight structural and insulating blocks [62,63]. The use of lightweight geopolymer aggregates also allows for the design of materials with controlled porosity and thermal conductivity, which makes it possible to optimize parameters for specific functional applications.

From the point of view of sustainable development, the technology of geopolymer concretes based on fly ash and industrial waste fits in with the principles of the circular economy, enabling both a reduction in CO_2_ emissions and the utilization of combustion by-products [64]. In combination with lightweight geopolymer aggregates, which can also be produced from waste materials, this technology opens up the prospect of producing low-emission, functional, new-generation construction materials.

The results obtained in this study are consistent with current trends in research on modified geopolymer composites and concretes containing functional additives. The literature has shown that the introduction of carbon nanomaterials, such as graphene, leads to a significant improvement in the resistance of geopolymers to high temperatures and the stability of their mechanical properties after thermal exposure, which is attributed to the sealing of the microstructure and the limitation of microcrack propagation [65]. In the context of the present study, this indicates that the observed thermal stability of lightweight geopolymer aggregates can be further enhanced through appropriate modification of the material composition. At the same time, studies on geopolymer composites modified with basalt fibers confirm their high fire resistance and ability to maintain structural integrity after exposure to elevated temperatures [66]. This indicates that materials of this type, including lightweight concretes containing porous aggregates, can be effectively used in components exposed to extreme thermal conditions, such as chimney liners. In turn, the use of industrial waste in cementitious and geopolymer composites not only affects their mechanical properties but also allows for their evaluation using non-destructive methods, which is of significant importance in the context of quality control and material durability [67]. These results confirm the validity of using fly ash as the main precursor for geopolymers and indicate the possibility of further optimizing properties through deliberate microstructure design. Additionally, it has been demonstrated that modifying concrete composites with fibrous additives and carbon-based materials leads to improved mechanical properties and load-bearing capacity of structural elements [68]. This indicates the potential for further development of lightweight geopolymer concretes through the use of hybrid material solutions combining lightweight aggregates with reinforcing additives. The results obtained are consistent with the current state of knowledge and confirm that lightweight geopolymer aggregates can serve as an effective component of concretes with enhanced thermal resistance and appropriate mechanical properties. At the same time, this study expands on previous research by focusing on a specific application—lightweight chimney liners—and integrating considerations of porosity, thermal insulation, and durability under high-temperature conditions.

In the future, further development of these materials may involve modifying the composition of the geopolymer matrix and aggregates to improve chemical durability, resistance to cyclic thermal loading, and compatibility with modern manufacturing technologies, such as modular prefabrication and 3D printing. A particularly promising direction is the design of multifunctional materials that combine structural load-bearing capacity with insulation, fire-resistant, and potentially environmental functions, which could significantly expand the range of applications for geopolymer concretes in modern construction and industry.

In addition to their attractive mechanical, thermal, and insulating properties, the studied lightweight geopolymer concretes also demonstrate significant potential for carbon dioxide (CO_2_) sequestration, which represents a promising direction for the development of these materials from both a scientific and an applied perspective. Literature findings and the authors’ observations to date indicate that alkali-activated materials, such as geopolymers, can effectively bind CO_2_ through carbonation reactions of the aluminosilicate matrix, leading to the permanent incorporation of CO_2_ into the material’s structure [69,70].

In the context of geopolymer concretes with lightweight aggregates, the porous structure of these materials—particularly in the case of foamed geopolymers and perlite—can promote increased reactive surface area and facilitate gas diffusion. The authors’ work on foamed geopolymers as insulating materials with CO_2_ adsorption capacity has shown that controlling porosity and microstructure leads to improved adsorption parameters, which creates the possibility of using these materials in processes of passive CO_2_ absorption from the environment [71,72].

CO_2_ sequestration in geopolymers can take both a chemical form, through the formation of stable carbonate complexes within the geopolymer structure, and a physical form, through adsorption in pores of appropriate size. This capability aligns with the concept of construction materials with enhanced environmental functionality, which, in addition to structural load-bearing capacity and temperature resistance, can also contribute to reducing CO_2_ concentrations in the local environment. This process is particularly significant in the context of global efforts to reduce greenhouse gas emissions and the pursuit of a low-carbon economy [73,74].

From a practical standpoint, the use of lightweight geopolymer concretes with CO_2_ sequestration capabilities can be applied in structures exposed to greenhouse gas emissions (e.g., industrial enclosures, transport tunnels, prefabricated building components) and as passive absorption elements in public spaces and storage facilities. Ultimately, the synergistic combination of mechanical, thermal, insulating, and environmental properties makes geopolymer lightweight materials a promising platform for future applications in sustainable construction and CO_2_ reduction technologies.

## 5. Conclusions

The conducted research allowed for the assessment of the impact of light mineral aggregates and foamed geopolymer aggregates on the mechanical and thermal insulation properties of geopolymer concretes intended for high-temperature applications. Based on the obtained results, the following conclusions were formulated:

1. Geopolymer concretes modified with lightweight mineral aggregates and foamed geopolymer aggregates can be effectively designed as structural and insulating materials for chimney components. The use of foamed geopolymer aggregates significantly reduces density and thermal conductivity while maintaining sufficient mechanical strength for the safe installation and operation of chimney systems.
2. The composition of mixtures significantly affects both the level of mechanical parameters obtained and their stability. The most balanced properties were obtained for mixtures containing a combination of foamed geopolymer aggregates and expanded clay, which combined moderate load-bearing capacity with low variability of results, which is important from the point of view of prefabrication of building elements.
3. An excessive increase in the amount of activating solution or the proportion of very light aggregates led to an increase in the dispersion of strength results, which indicates the need for precise control of the composition of mixtures and the forming process. At the same time, the very low thermal conductivity values of foamed geopolymer aggregates (≈0.075 W/(m·K)) confirm their high potential as a functional thermal insulation filler.
4. The results obtained confirm that geopolymer concretes with lightweight mineral and geopolymer aggregates can be a durable and low-emission alternative to traditional ceramic and expanded clay solutions in modern chimney systems. and geopolymer aggregates can be a durable and low-emission alternative to traditional ceramic and expanded clay solutions in modern chimney systems, representing a step towards the design of single-layer, lightweight elements combining load-bearing and insulating functions.

## Figures and Tables

**Table 1:** Fly ash, sand, and metakaolin–oxide analysis.

Precursor		Oxide Composition (wt.%)					
	SiO_2_	Al_2_O_3_	Fe_2_O_3_	CaO	K_2_O	TiO_2_	SO_3_
**Fly ash**	58.28	31.90	3.85	2.29	2.05	0.76	0.58
**Sand**	98.73	–	0.23	0.15	0.48	0.02	0.19
**Metakaolin**	51.90	46.17	0.66	0.26	0.70	0.19	–

**Table 2:** Fly ash, sand, and metakaolin–particle size distribution.

Material	D_10_ [μm]	D_50_ [μm]	D_90_ [μm]	Average Value [μm]	Standard Deviation [μm]
**Fly ash**	2.26	12.36	31.75	16.10	0.03
**Sand**	257.64	360.71	483.04	406.28	0.02
**Metakaolin**	1.61	8.95	27.24	12.67	0.02

**Table 3:** Identification of the tested samples and their material composition.

ID	Fly Ash [kg/m^3^]	Sand [kg/m^3^]	Perlite [kg/m^3^]	GAFA [kg/m^3^]	GAM [kg/m^3^]	KER [kg/m^3^]	Activator [kg/m^3^]
GEO3.25	254.1	254.1	21.2	84.7	0.0	0.0	195.4
GEO3.35	253.0	253.0	21.1	84.3	0.0	0.0	200.7
GEO3.5	251.4	251.4	20.9	83.8	0.0	0.0	208.3
2GEO3.25	254.1	254.1	21.2	42.3	42.3	0.0	195.4
2GEO3.5	251.4	251.4	20.9	41.9	41.9	0.0	208.3
1.5KER3.25	256.6	256.6	21.4	0.0	0.0	64.2	197.4
2KER3.25	239.0	239.0	19.9	0.0	0.0	79.7	183.9
KERGEO3.25	258.3	258.3	21.5	8.6	0.0	56.0	198.6
2KERGEO3.25	246.3	246.3	20.5	41.1	0.0	41.1	189.4

**Table 4:** Average thermal conductivity values for geopolymer concretes with lightweight aggregates.

Sample ID	Average Thermal Conductivity Coefficient [W/m⋅K]	Standard Deviation [W/m⋅K]
GEO3.25	0.07596	0.00001
GEO3.35	0.09499	0.00001
GEO3.5	0.08782	0.00001
2GEO3.25	0.09192	0.00571
2GEO3.5	0.07523	0.00001
1.5KER3.25	0.09650	0.01126
2KER3.25	0.09947	0.00001
KERGEO3.25	0.08947	0.00001
2KERGEO3.25	0.08900	0.00035

**Table 5:** Average compressive strengths of the tested geopolymer concretes with lightweight aggregates.

Sample ID	Average Compressive Strength [MPa]	Standard Deviation [MPa]
GEO3.25	29.63	5.96
GEO3.35	10.40	1.98
GEO3.5	18.47	6.74
2GEO3.25	15.08	2.19
2GEO3.5	13.55	2.83
1.5KER3.25	14.41	1.57
2KER3.25	13.30	1.98
KERGEO3.25	8.70	1.13
2KERGEO3.25	16.38	1.20

**Table 6:** Average flexural tensile strengths of the tested geopolymer concretes with lightweight aggregates.

Sample ID	Average Flexural Tensile Strengths [MPa]	Standard Deviation [MPa]
GEO3.25	3.85	0.51
GEO3.35	2.35	0.07
GEO3.5	3.20	0.85
2GEO3.25	2.63	0.25
2GEO3.5	2.55	0.41
1.5KER3.25	2.91	0.39
2KER3.25	2.80	0.01
KERGEO3.25	2.20	0.28
2KERGEO3.25	2.90	0.14

## Data Availability

The original contributions presented in this study are included in the article. Further inquiries can be directed to the corresponding authors.
